# Platelet distribution width, mean platelet volume and haematological parameters in patients with uncomplicated *plasmodium falciparum* and *P. vivax* malaria

**DOI:** 10.12688/f1000research.11767.1

**Published:** 2017-06-12

**Authors:** Elrazi A. Ali, Tajeldin M. Abdalla, Ishag Adam

**Affiliations:** 1 Faculty of Medicine, University of Khartoum, Khartoum, Sudan; 2 Faculty of Medicine, Kassala University, Kassala, Sudan

**Keywords:** hematological profile, PDW, MPV, plasmodium falciparum, P. vivax

## Abstract

Background: The association between the haematological profile (including abnormal platelets) and malaria is not completely understood. There are few published data on haematological profiles of malaria patients in areas with unstable malaria transmission. The current study was conducted to investigate if the haematological parameters, including platelet indices, were reliable predictors for microscopically-diagnosed malaria infection.

Methods: A case-control study with a total of 324 participants (162 in each arm) was conducted at the out-patient clinic of New Halfa hospital during the rainy and post rainy season (August 2014 through to January 2015). The cases were patients with uncomplicated *Plasmodium falciparum* (107; 66.9%) and *P. vivax* malaria (55, 34.0%) infections. The controls were aparasitemic individuals. The haematological parameters were investigated using an automated hemo-analyser.

Results: There was no significant difference in the mean (±SD) age between the study groups; however, compared to the controls, patients with uncomplicated malaria had significantly lower haemoglobin, leucocyte and platelet counts, and significantly higher red cell distribution width (RDW), platelet distribution width (PDW) and mean platelet volume (MPV).

Conclusions: The study revealed that among the haematological indices, PDW and MPV were the main predictors for uncomplicated *P. falciparum* and P *. vivax *malaria infection *.*

Abbreviations: OR: odds ratio.

## Introduction

In spite of the preventative measures, malaria remains a major public health concern. Malaria is responsible for 781,000 deaths in a year, the majority of which are in Sub -Saharan Africa ^1^. A correct diagnosis is one of the most important tools in the management of malaria. It has been recommended that all persons with suspected malaria should have a parasitological confirmation of diagnosis ^1^. Microscopic examination of malaria consists of the identification of parasite species in thin and/or thick blood films, which is the “gold standard” for malaria diagnosis ^1, 2^. Microscopy requires trained technicians, and well-maintained microscopes with a perfect quality management system. However, acceptable microscopy services are not widely available for the diagnosis of malaria in some areas where malaria is endemic e.g. in communities in Sub-Saharan Africa ^1^.

Previously, measurement of haematological blood parameters was unreliable due to intra and inter-method variation. Nowadays, automated analysers have replaced the traditional methods. Automated analysers are available in most settings and can give reliable results within a short period of time. There is a universal trend toward using these to aid the presumptive diagnosis of malaria infection ^2, 3^.

Previous studies have reported different results levels of sensitivity and specificity of haematological parameters as predictors of malaria infection ^4– 8^. There is no published data on haematological changes in patients infected with malaria parasites in Sudan, where malaria in the major health problem ^9^. The current study was conducted in New Halfa, eastern Sudan to investigate the haematological changes observed during malaria infection and to assess the reliability of the haematological parameters used for diagnosis.

## Methods

A case-control study was conducted at the out-patient clinic of New Halfa hospital during the rainy and post rainy season (August 2014 through to January 2015). The cases were patients with symptoms and signs of uncomplicated malaria and who were confirmed to be infected with *P. falciparum or P. vivax* by microscopic examination of Giemsa stained blood smears during the study ^10^. The controls were the patients that presented to the same clinic with symptoms of malaria but were found to have negative blood films for malaria. After the participants (or their parents/legal guardians if they were minors) provided written informed consent, a clinical history was gathered using questionnaires. Weight and height were measured and body mass index was expressed as kg/m ^2^.

2ml of blood was taken from each participant and placed in a container with EDTA, and a complete hemogram was performed using an automated hematology analyser (Sysmex XN-9000; Hyogo, Japan), following the manufacturers' instructions as previously described ^11– 13^. The hemogram included measuring the haemoglobin level, leucocyte count and platelet indices, namely platelet count, mean platelet volume (MPV), and platelet distribution width (PDW).

Thick and thin blood films were prepared and stained with 10% Giemsa to microscopically confirm which participants were infected. If the slide was positive, the parasite density was measured by counting the number of asexual parasites per 200 leukocytes, and multiplied against the participants own leucocytes number/μL. The blood films were considered negative if no parasites were detected in 100 oil immersion fields of a thick blood film.

### Statistical analysis

A minimum sample size of 162 participants for each arm of the study was calculated assuming that 10% of participants would have incomplete data. In this way, it would be possible to calculate a significant difference (at α = 0.05) in the means of the proposed variables - mainly haemoglobin, red cell distribution width (RDW), leucocytes, platelets counts and PDW - between the cases and the controls, at 80% power.

Statistical analysis was performed using SPSS for Windows, version 20.0 (SPSS Inc., Chicago, IL, USA). Proportions of the studied groups were expressed as percentages and compared using the chi-squared test. Continuous data were checked for normality using the Shapiro-Wilk test. The means (±SD) or median (IQR) were used to describe the studied variables, depending if they were normally or non-normally distributed. The t-test (or Mann-Whitney U test if the data were not normally distributed) evaluated the differences between the studied groups. Binary regression was calculated, where malaria was the dependent variable and medical and haematological indices were the independent variables. Diagnostic screening tests were used to determine the diagnostic cut-offs of various parameters (based on test sensitivity and specificity) using the receiver operating characteristic (ROC) curve. P < 0.05 was considered statistically significant.

## Results

107 (66.9%) and 55 (34.0%) of the uncomplicated malaria cases were infected with *P. falciparum* and *P vivax*, respectively. There was no significant difference in the age or BMI between the cases and the controls. Patients had significantly higher body temperature than the controls ( Table 1). Their ages ranged between 1.1−55 years in the cases and 1.1−42 years in the controls. Around one third of the cases (53, 32.7%) and one third of the controls (49, 30.2%) were children that were under five years old (p=0.665). There were 81 (50.0%) vs. 79 (48.8%) males in the cases and controls, respectively (p=0.912).

**Table 1.** Comparing the mean (±SD) age, BMI and body temperature between patients with malaria and negative controls.

Variables	Patients with malaria (n=162)	Controls (n=162)	P-value
Age, years	20.7(19.6)	20.0 (19.0)	0.738
Body mass index, kg/m ^2^	19.8(6.2)	19.9(14.3)	0.947
Temperature	38.0 (0.9)	37.6(1.0)	0.002

Compared with the controls, patients with uncomplicated malaria had significantly lower haemoglobin levels and lower leucocyte, lymphocyte, neutrophil, and platelet counts, but significantly higher RDW, PDW and MPV ( Table 2).

**Table 2.** Comparing the median (IQR) of the blood parameters measured in patients with and without malaria. Data is displayed as mean (±SD), and the t-test was used because the data was normally distributed.

Variables	Patients with malaria (n=162)	Controls (n=162)	P-value
Hemoglobin, g/dl*	9.3(2.3)	10.2 (2.0)	0.003
Red cell distribution width,%	16.0(14.2−19.1)	15.1 (13.6−18.3)	0.034
Leucocytes, X10 ^3^/mm ^3^	6.5(5.2−8.4)	8.2 (5.65−10.37)	0.009
Lymphocytes, X10 ^3^/mm ^3^	2.448 (1.668−3.501)	2.53 (1.70−4.03)	0.041
Neutrophils, X10 ^3^/mm ^3^	3.209(2.185−4.686)	4.64 (2.77−6.31)	0.003
Platelet count, X10 ^3^/mm ^3^	121.(78.0−186.5)	280.0 (227.0−350.0)	< 0.001
Platelets distribution width, %	14.7(14.4−15.0)	14.5 (14.3−14.8)	0.003
Mean platelet volume, fL	9.6(9.2−10.0)	8.8 (8.5−9.4)	< 0.001

**Figure 1.**
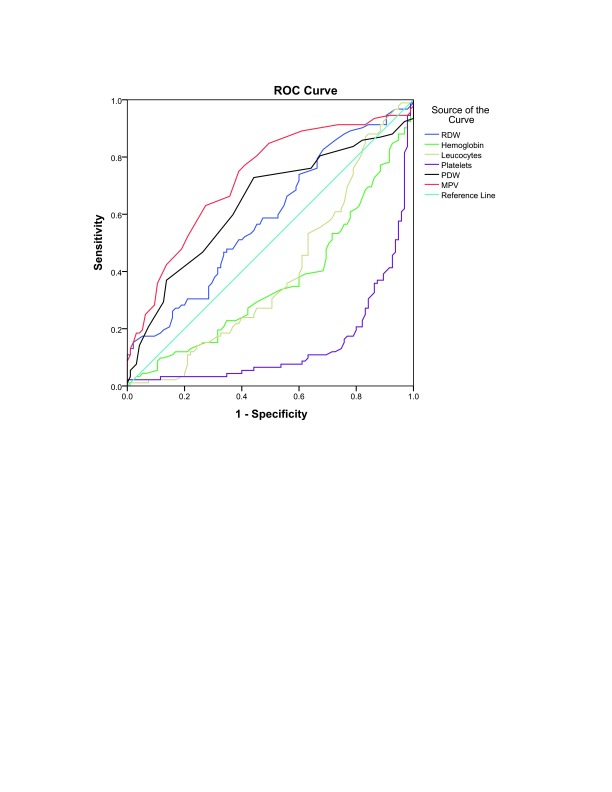
Receiver operating characteristic (ROC) curve and the associated reliability of blood parameters in predicting malaria in eastern Sudan.

A receiver operating characteristic (ROC) curve was used to determine the cut-offs for haemoglobin levels, RDW, leucocytes and platelet counts, PDW and MPV for prediction of malaria infection. The area under the ROC curve is shown in Table 3 and Figure 1, which failed to confirm predictability of hemoglobin, RDW, leucocytes and platelet count. Poor and fair predictability of PDW and MPV for malaria infection was demonstrated; the areas under the curves were 0.637 and 0.726, respectively.

**Table 3.** The reliability of haematological indices in predicting malaria.

Variable	Area under the curve	P-value	Sensitivity	Specificity	Cut-off
Hemoglobin, g/dl ^*^	0.365	0.001			
Red cell distribution width, %	0.587	0.039			
Leucocytes, X10 ^3^/mm ^3^	0.396	0.014			
Platelet count, X10 ^3^/mm ^3^	0.143	< 0.001			
Platelets distribution width, % ^*^	0.637	0.001	72.8	56.8	14.550
Mean platelet volume, fL ^*^	0.726	0.000	77.2	60.1	9.05

Key: ^*^: These were considered because of fair predictability of the area under the curve

When the cut-off levels were evaluated using binary regression analysis, PDW ≥14.550 % (OR =2.9, 95% CI =1.64−5.43, P < 0.001) and MPV≥ 9.05fL (OR =2.25, 95% CI =1.12−4.51, P < 0.001) were the most important predictors for malaria infection, ( Table 4).

**Table 4.** Binary logistic regression analysis of the factors associated with *P. falciparum* and *P. vivax* malaria in eastern Sudan. OR: odds ratio.

Variable	OR	95% CI	P-value
Age	0.99	0.93−1.06	0.986
Male gender	0.93	0.39−2.19	0.870
Body mass index	1.09	0.94−1.26	0.222
Temperature	1.33	0.87−2.03	0.185
Hemoglobin	0.90	0.72−1.13	0.382
Red cell distribution width	1.16	0.99−1.36	0.054
Leucocytes	0.96	0.84−1.10	0.605
Platelets count	0.98	0.98−0.99	0.000
Platelets distribution width ≥14.550 %	2.9	1.64−5.43	< 0.001
Mean platelets volume ≥ 9.05fL	2.25	1.12−4.51	< 0.001

There was no significant difference in the hemoglobin, leucocytes, lymphocytes, neutrophils, platelets counts, RDW, PDW, MPV and the parasite count (P=0.201) when the cases of *P. falciparum* and *P. vivax* were compared ( Table 5).

**Table 5.** Comparing the median (IQR) blood parameters in patients with *P. falciparum* and *P. vivax* malaria. Data is displayed as mean (±SD), and the t-test was used because the data was normally distributed.

Variables	Patients with *P. falciparum* malaria (n=107)	Patients with *P. vivax* malaria (n=55)	P
Hemoglobin, g/dl ^*^	9.3(2.3)	10.2 (2.0)	0.003
Red cell distribution width,%	15.5 (14.1−19.0)	17.1(14.1−19.8)	0.225
Leucocytes, X10 ^3^/mm ^3^	6.5 (5.1−8.1)	6. 4 (5.1−9.5)	0.504
Lymphocytes, X10 ^3^/mm ^3^	2.454 (1.654−3.373)	2.43 (1.82−3.95)	0.677
Neutrophils, X10 ^3^/mm ^3^	3.090 (1.958−4.389)	3.27 (2.38−5.31)	0.641
Platelet count, X10 ^3^/mm ^3^	115.0 (70.5−183.5)	133.5 (98.7−205.7)	0.168
Platelets distribution width, %	14.8 (14.5−15.0)	14.6 (14.2−14.9)	0.186
Mean platelets volume, fL	9.7 (9.1−10.1)	9.4(9.1−9.9)	0.366

Raw data collected as the basis for this study Plasmf = Blood film for *P. falciparum*. Click here for additional data file. Copyright: © 2017 A. Ali E et al. 2017 Data associated with the article are available under the terms of the Creative Commons Zero "No rights reserved" data waiver (CC0 1.0 Public domain dedication).

## Discussion

According to our present findings, PDW and MPV are the two most important haematological predictors of *P. falciparum* and *P. vivax* malaria infection. This is in line with a recent finding where Al-Salahy *et al.* reported that patients in Hajjah, Northwest Yemen with malaria parasitemia had significantly lower hemoglobin, hematocrit, leucocytes, lymphocytes, and platelet counts compared to healthy subjects ^14^. Previous studies have shown that patients with complicated malaria had reduced haematological parameters such as platelet, leucocyte, and RBC counts, which provided relatively good predictors for the diagnosis of malaria infection ^8, 15^. On the other hand, the significant differences observed in the haematological parameters between parasitemic Ugandan patients and non-parasitemic Ugandans were only observed in the monocyte and the platelet count ^16^. No significant difference was found between the haemoglobin levels, MCV, MCH, neutrophils, lymphocyte counts or MPV ^16^.

In the current study, a PDW ≥14.550% and MPV ≥ 9.05fL were the main predictors for malaria (OR =2.9 and 2.3). Previous studies have reported an increased MPV level in malaria ^15, 17^. Interestingly, Chandra *et al* reported that an MPV > 8 fL had a sensitivity and specificity of 70.8% and 50.4% for the diagnosis of malaria, respectively ^8^.

The higher PDW and MPV values in malaria could be explained by bone marrow formation of megakaryocytes to compensate for the low absolute platelet count during acute malaria infection ^8, 15^. A significantly higher level of the key platelet growth factor (thrombopoietin) has been reported in patients with malaria ^18^. Furthermore, the parasitized RBCs could increase in platelet sensitivity to adenosine diphosphate (ADP), prompting secretion of dense granules ^19, 20^.

Nutritional deficiency and haemoglobinopathies were not investigated in the current study and have to be mentioned as study limitations. Haematological parameters formalaria-infested blood may vary depending on the level of malaria endemicity, presence of haemoglobinopathies and nutritional status ^21, 22^. Another limitation of the is that we relied on microscopy only for the malaria diagnosis. Some of negative controls may have had undetected parasitemia (submicroscopic parasitemia). We have previously observed that the majority of febrile patients who were parasite negative by microscopy had *P. falciparum* infection according to PCR results ^23^. Lastly, other infections that might have an effect on blood parameters were not ruled out in both the cases and controls. In conclusion, the study revealed that a PDW ≥14.550% and MPV ≥ 9.05fL were the main predictors for uncomplicated *P. falciparum* and *P. vivax* malaria infection.

## Data availability

The data referenced by this article are under copyright with the following copyright statement: Copyright: © 2017 A. Ali E et al.

Data associated with the article are available under the terms of the Creative Commons Zero "No rights reserved" data waiver (CC0 1.0 Public domain dedication). 

**Dataset 1: Raw data collected as the basis for this study.** Plasmf = Blood film for *P. falciparum*. DOI, 10.5256/f1000research.11767.d164010 ^24^

## Consent

The study was approved by the Institutional Review Board of the Medical College, University of Khartoum (3# 2015 1114).
