# Supplementary material for: Platelet distribution width, mean platelet volume and haematological parameters in patients with uncomplicated plasmodium falciparum and P. vivax malaria
Source: F1000Res. 2017 Jun 12;6:865. [Version 1] doi: 10.12688/f1000research.11767.1 (PMC5538039; doi:10.12688/f1000research.11767.1)
Supplement: Peer review history

# Peer review: Referee response for version 1

**DOI:** 10.5256/f1000research.12711.r23927

**Published:** 2017-07-31

**Authors:** Walter R.J. Taylor

**Affiliations:** 1 Mahidol Oxford Tropical Medicine Research Unit, Bangkok, Thailand

This is a nice academic paper that deals with haematological parameters in malaria. Using a case control approach, the authors have identified a mean platelet distribution width and mean platelet volume as possible markers of acute symptomatic malaria in their setting.

 This is interesting but of limited practical application. Therefore, this paper should emphasise the academic aspects of their findings.  

 I think this paper can be accepted but with major revisions in two areas, in particular.

 Sample size calculation – this has been determined using continuous variables yet we do not see any examples. This must be addressed.

 Discussion – this should be more thorough comparing and contrasting the current data with previously published data. Moreover, readers will want to know important causes of increased RDW, PDW and MPV and where in haematological practice these parameters play an important role.

 The authors should let us know if their research has led to any new research questions and future avenues of research. 

 Minor comments

- The number of malaria related deaths are higher than those reported by the WHO. The authors should obtain the latest data.
- No mention of the role of malaria rapid diagnostic tests
- The second line of the Discussion does not follow. Did the Yemeni study report PDW and MPV ?
- Page 6 left hand column penultimate paragraph:
  Remove 'in' in this sentence:
  Furthermore, the parasitized RBCs could increase in platelet sensitivity to adenosine diphosphate (ADP), prompting secretion of dense granules. 

I have read this submission. I believe that I have an appropriate level of expertise to confirm that it is of an acceptable scientific standard, however I have significant reservations, as outlined above.

# Peer review: Referee response for version 1

**DOI:** 10.5256/f1000research.12711.r23451

**Published:** 2017-07-11

**Authors:** Alberto Tobón-Castaño

**Affiliations:** 1 Malaria Group, Faculty of Medicine, University of Antioquia, Medellín, Colombia

1. The objective of the study should be re-examined in the introduction. The terms "reliability" and "validity" should be differentiated. Reliability refers to the degree to which the measurement procedure can be reproduced.
2. Thus, if the objective is to study the validity of the hematological parameters to make a diagnosis, the methodology must be revised since these studies require a sample size that considers the prevalence and the expected sensitivity. Since this is a case-control study the objective should be consistent with this methodology.
3. It must be specified what a case is; What are the signs and symptoms of uncomplicated malaria?
4. It should be explained why 100 oil inmmersion fields were used as criteria to consider a thick blood film as negative. This does not correspond to international recommendations.
5. The calculation of the sample should be presented with the elements of a case-control study i.e. case-control relation, expected O.R., frequency of outcomes in exposed and unexposed.
6. RESULTS. If the clinical history is available, the time of evolution of the disease could be compared, which may lead to differences in hematological profiles.
7. The results presented in the Tables 2 and 5 are confusing. It is not clear whether the averages or medians are compared and what was the statistical test of comparison. It is not explicitly stated which variables presented normal distribution.
8. The presumptive diagnosis of malaria with haematological analyzers does not seem to be the appropriate way of solving malaria diagnosis problems in endemic areas. In this sense, it is not clear whether the study intends to contribute to the malaria diagnosis using a method that would be non-specific.

The study contributes to show hematological differences between cases and controls and this should serve to identify early lesions, but clearly is no a useful method for malaria diagnosis.

 There is no relevant discussion about the findings. The reference values of the hematological parameters according to age and sex are not presented; the comparisons must take into account this variation.

 Some aspects related to platelet changes are analyzed but superficially. Leukocyte count and cell line analysis are neglected; no mention is made of the significance of alterations, monocytes and eosinophils are neglected. There are no correlations with parasitemias which is also a variable that can contribute significantly to the hematological alterations as a function of the greater or lesser inflammatory response.

 REFERENCES.

 Reference 1.  Who are the authors?

 Ref 6 and 8 are the same.

 Ref. 16.  The titlle is incomplete? The journal name?

I have read this submission. I believe that I have an appropriate level of expertise to confirm that it is of an acceptable scientific standard, however I have significant reservations, as outlined above.

# Peer review: Referee response for version 1

**DOI:** 10.5256/f1000research.12711.r23925

**Published:** 2017-07-07

**Authors:** Con J.F. Fontes

**Affiliations:** 1 Laboratório de Malária, Hospital Universitário Júlio Müller, Rua Prof Luiz Phelippe Pereira Leite s/n Alvorada, Cuiabá, Brazil

Mr Ali and colleagues present a study that revealed the mean platelet volume and platelet distribution width as good parameters to predict uncomplicated malaria parasite infection.The manuscript is well written and presented. The analysis of the results is adequate and correct. Some minor points should be reviewed:

1. As the authors used several statistical tests to compare the results between cases and controls, it is important to make these tests explicit for each p-value presented in the tables, as a footnote.
2. The title of Table 5 is not clear. In fact, Table 5 compares means (SD), and non-median (IQR).
3. In the last paragraph of the Discussion section the sentence "...parameters formalaria-infested blood..."  should be changed by "... for malaria-infected blood...".

I have read this submission. I believe that I have an appropriate level of expertise to confirm that it is of an acceptable scientific standard.
